# Supplementary material for: Surface Heterogeneous Nucleation-Mediated Release of Beta-Carotene from Porous Silicon
Source: Nanomaterials (Basel). 2020 Aug 24;10(9):1659. doi: 10.3390/nano10091659 (PMC7560142; doi:10.3390/nano10091659)
Supplement: Supplementary file 1 [file nanomaterials-10-01659-s001.pdf]

nanomaterials

Supplementary Material

Surface Heterogeneous Nucleation-Mediated Release of Beta-Carotene from Porous Silicon

Chiara Piotto [1], Sidharam P. Pujari [2], Han Zuilhof [2,3,4] and Paolo Bettotti [1,*]

[1] Nanoscience Laboratory, Department of Physics, University of Trento, 38123 Povo, Trento, Italy; chiara.piotto@gmail.com
[2] Laboratory of Organic Chemistry, Wageningen University, Stippeneng 4, 6708 Wageningen, The Netherlands; sidharam.pujari@wur.nl (S.P.P.); han.zuilhof@wur.nl (H.Z.)
[3] Department of Chemical and Materials Engineering, Faculty of Engineering, King Abdulaziz University, 21589 Jeddah,
Saudi Arabia
[4] School of Pharmaceutical Sciences and Technology, Tianjin University, 92 Weijin Road, Tianjin 300072, China
* Correspondence: paolo.bettotti@unitn.it; Tel.: +39-0461-281501

Section S1. Evaluation of the Homogeneity of the Impregnation

Since the method we used to load PSi samples requires the sequential deposition of some liquid drops on their surface to achieve the desired loading, we might expect the formation of coffee-stain on the edge of the porous area. To evaluate it, we loaded a PSi sample and we cleaved it along the blue dotted lines sketched in Figure S1.

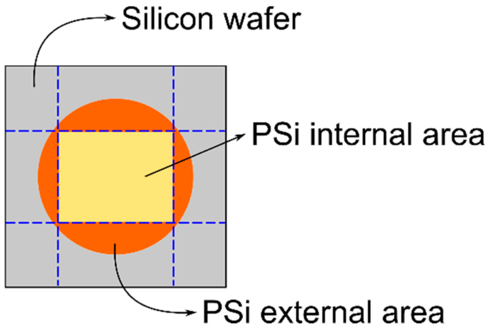

Figure S1. Schematic of PSi internal and external areas.

Then, we release the betacarotene (BCAR) in tetrahydrofuran (which is an excellent solvent for BCAR) separately for the external part (55% of the total area, orange area in Figure S1) and for the internal one (45% of the total area, yellow area in Figure S1). We estimate a negligible effect of the coffee stain since 52% of the BCAR was released from the external area and 48% from the internal one.

Section S2. Pore Size Evaluation of the PSi-B from SEM Top View

Since the branched shape of the pores of PSi-B sample, their diameter has been estimated from SEM top view. This method is slightly less accurate, than looking at the cross section, as it cannot detect the deviation from the average pore size that often affect the PSi superficial layer. Yet, the results obtained confirm that both PSi-A and PSi-B have similar pore size.

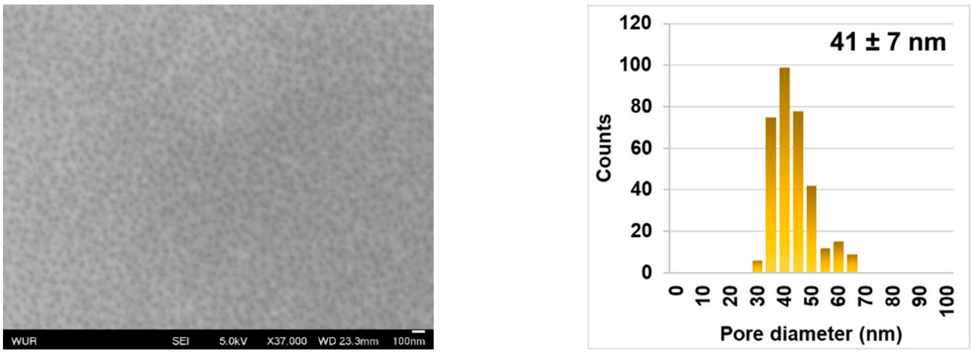

(a) (b)

Figure S2. (**a**) SEM top view of the PSi surface without BCAR loaded, (**b**) histogram of the pore size statistic. The average pore size is 41 ± 7 nm (n = 336 pores).

© 2020 by the authors. Licensee MDPI, Basel, Switzerland. This article is an open access article distributed under the terms and conditions of the Creative Commons Attribution (CC BY) license (http://creativecommons.org/licenses/by/4.0/).
